# Supplementary material for: Risk Factors Associated with Pancreatic Cancer in the UK Biobank Cohort
Source: Cancers (Basel). 2022 Oct 12;14(20):4991. doi: 10.3390/cancers14204991 (PMC9599736; doi:10.3390/cancers14204991)
Supplement: Supplementary file 1 [file cancers-14-04991-s001.zip › cancers-1906797-supplementary.pdf]

Table S1. Codes used to identify pancreatic cancer (PaCa) cases and non-pancreatic cancer controls.

Categories	Frequency (%)	ICD10 codes	ICD9 codes	Self-reported cancer codes
**Pancreatic cancer cases**				
Incident cases	728 (0.14%)	Codes start with C25 and its subclasses, C25.0, C25.1, C25.2, C25.3, C25.4, C25.7, C25.8, and C25.9	Codes start with 157 and its subclasses 1570, 1572, 1574, and 1579	1026 code only
**Non-pancreatic cancer control**s				
All healthy controls	412,922 (82.19%)	Subjects with no code assigned	Subjects with no code assigned	Subjects with no code assigned
**Subjects excluded from the study**				
1- Other neoplasms (included in situ neoplasms, benign neoplasms and neoplasm of unknown nature or behavior)	88,672 (17.65%)	Codes start with C (C00-C97) except codes for PaCa, and codes start with D (D00-D48)	Codes 1140-239 except codes for PaCa	All other codes except the 1026 code
2- Prevalent cases	91 (0.02%)	Codes start with C25 and its subclasses, C25.0, C25.1, C25.2, C25.3, C25.4, C25.7, C25.8, and C25.9	Codes start with 157 and its subclasses 1570, 1572, 1574, and 1579	1026 code only
**Total**	502,413 (100%)			

Table S2. Classification of the exposure variables included in the analysis.

	Variable	Groups	Coding
1.	Gender	Female	This variable is described as the biological sex provided by the UK Biobank (reference field ID: 31).
		Male	
2.	Age	Continuous data	The age in the case group is described as the age diagnosed as PaCa (reference field ID: 40008), and the age in the control group is calculated till the loss of follow-up date (reference field ID:21003).
3.	Ethnic group	White	This variable is described as the ethical background information provided by the UK Biobank (reference field ID: 21000).
		Mix	
		Asian	
		Black	
		Chinese	
		Other	
4.	Smoking (including cigarettes, pipes, cigars, etc.)	Never	This variable is described as the smoking status information provided by the UK Biobank (reference field ID: 20116).
		Previous	
		Current	
5.	Cigarette smoking	Never	Cigarette smoking is recognized by the type of currently tobacco smoked, the number of cigarettes previously smoked daily and the number of cigarettes currently smoked daily from the UK Biobank (reference field ID: 3446, 2887 and 3456).
		Previous	
		Current	
6.	Alcohol	Never	This variable is described as the alcohol intake frequency provided by the UK Biobank (reference field ID: 1558).
		Ocassions,1-3 times/m	
		1-4 times/w	
		Daily	
7.	BMI	Normal or underweight (BMI<25)	The BMI information is continuous data initially provided by the UK Biobank (reference field ID:21001). The classification was divided into three groups based on NHS suggestion.
		Overweight (25 ≤ BMI < 30)	
		Obese (BMI ≥ 30)	
8.	Waist Circumference (cm)	Continuous data	This variable is continuous data provided by the UK Biobank (reference

			field ID: 48).
9.	Waist Hip Ratio	Normal (M: <0.90, F: <0.85)	This variable is initially calculated by dividing the waist circumference over the hip circumference (reference field ID:49) and then categorized into the normal group and abdominal obesity group according to WHO recommendation.
		Abdominal obesity (M: ≥0.90, F: ≥0.85)	
10.	Processed meat intake	Never	This variable is described as the proceeded meat intake provided by the UK Biobank (reference field ID:1349).
		<1 time/ week	
		1 time/ week	
		2-4 times/week	
		5-6 times/week	
		≥ 1 time/ day	
11.	Pancreatitis	Yes or No	This variable is obtained by using self-reported non-cancer illness medical condition (reference field ID: 20002) to identify pancreatitis code 1165.
12.	Diabetes Mellitus	Yes or No	This variable is obtained by using self-reported non-cancer illness medical conditions (reference field ID: 20002) to identify Diabetes Mellitus codes 1220,1222, and 1223.
13.	Hepatitis B	Yes or No	This variable is obtained by using self-reported non-cancer illness medical condition (reference field ID: 20002) to identify Hepatitis B code 1579.
14.	Cholecystitis	Yes or No	This variable is obtained by using self-reported non-cancer illness medical condition (reference field ID: 20002) to identify cholecystitis code 1579.
15.	Helicobacter pylori infection	Yes or No	This variable is obtained by using self-reported non-cancer illness medical condition (reference field ID: 20002) to identify Helicobacter pylori infection code 1442.
16.	Systemic lupus erythematosus	Yes or No	This variable is obtained by using self-reported non-cancer illness medical condition (reference field ID: 20002) to identify Systemic lupus erythematosus code 1381.
